# Daily Health and Well-Being Among Caregivers With Multiple Adverse Childhood Experiences

**DOI:** 10.1093/geroni/igaf122.2109

**Published:** 2025-12-31

**Authors:** Yin Liu, Jooyoung Kong, David Almeida, Stephanie Robert

**Affiliations:** Utah State University, Logan, Utah, United States; University of Wisconsin–Madison, Madison, Wisconsin, United States; The Pennsylvania State University, University Park, Pennsylvania, United States; University of Wisconsin–Madison, Madison, Wisconsin, United States

## Abstract

**Background/objective:**

Caregiving for aging parents is a prevalent experience for middle- and older adults in the US. Utilizing a life course perspective to family caregiving, this study examined the associations between providing care to parents and daily well-being outcomes, while also testing the moderating roles of adverse childhood experiences (ACEs) and current family support or strain.

**Methods:**

Data were drawn from the National Study of Daily Experiences (NSDE) 3, a series of daily diary surveys over eight consecutive days. A study sample included 434 caregivers and their 1,123 daily diary records. The daily outcomes included daily negative and positive affect, physical symptoms, and sleep quality. A multilevel modeling approach was employed to account for days nested within individuals.

**Results:**

Having three or more ACEs significantly moderated the within-person association between caregiving for parents and higher daily negative affect. Among respondents with three or more ACEs, positive family support buffered the associations between daily caregiving to parents and higher negative affect, lower positive affect, and poorer sleep quality. Family strain exacerbated the effects of daily caregiving to parents on higher negative affect, lower positive affect, more physical symptoms, and poorer sleep quality.

**Conclusions:**

ACEs play a crucial role in contextualizing caregivers’ daily health outcomes. This study highlights the importance of trauma-informed support tailored to these caregivers who have faced multiple ACEs. Future research should focus on exploring the specific coping mechanisms and cumulative disadvantages of these caregivers to inform more effective, targeted interventions.

